# Machine Learning Model for Predicting Acute Respiratory Failure in Individuals With Moderate-to-Severe Traumatic Brain Injury

**DOI:** 10.3389/fmed.2021.793230

**Published:** 2021-12-24

**Authors:** Rui Na Ma, Yi Xuan He, Fu Ping Bai, Zhi Peng Song, Ming Sheng Chen, Min Li

**Affiliations:** ^1^Department of Pulmonary and Critical Care Medicine, The Second Affiliated Hospitals of Fourth Military Medical University, Xi'an, China; ^2^Neurocritical Care Unit, Department of Neurosurgery, The Second Affiliated Hospitals of Fourth Military Medical University, Xi'an, China; ^3^Department of Neurosurgery, Lin Fen Center Hospital, Lin Fen, China

**Keywords:** acute respiratory failure, machine learning, XGBoost model, logistic regression, traumatic brain injury

## Abstract

**Background:** There is a high incidence of acute respiratory failure (ARF) in moderate or severe traumatic brain injury (M-STBI), worsening outcomes. This study aimed to design a predictive model for ARF.

**Methods:** Adult patients with M-STBI [3 ≤ Glasgow Coma Scale (GCS) ≤ 12] with a definite history of brain trauma and abnormal head on CT images, obtained from September 2015 to May 2017, were included. Patients with age >80 years or <18 years, multiple injuries with TBI upon admission, or pregnancy (in women) were excluded. Two models based on machine learning extreme gradient boosting (XGBoost) or logistic regression, respectively, were developed for predicting ARF within 48 h upon admission. These models were evaluated by out-of-sample validation. The samples were assigned to the training and test sets at a ratio of 3:1.

**Results:** In total, 312 patients were analyzed including 132 (42.3%) patients who had ARF. The GCS and the Marshall CT score, procalcitonin (PCT), and C-reactive protein (CRP) on admission significantly predicted ARF. The novel machine learning XGBoost model was superior to logistic regression model in predicting ARF [area under the receiver operating characteristic (AUROC) = 0.903, 95% CI, 0.834–0.966 vs. AUROC = 0.798, 95% CI, 0.697–0.899; *p* < 0.05].

**Conclusion:** The XGBoost model could better predict ARF in comparison with logistic regression-based model. Therefore, machine learning methods could help to develop and validate novel predictive models.

## Introduction

Acute respiratory failure (ARF) is a common pathophysiological result of pulmonary complications [pneumonia, neurogenic pulmonary edema, and acute respiratory distress syndrome (ARDS)] in moderate or severe traumatic brain injury (M-STBI), not only worsening outcomes, but also extending intensive care unit (ICU) and hospital stays and increasing the cost of hospital care (1–7). Consequently, accurately predicting ARF risk may help to identify cases requiring intensive airway management. This would help to allocate resources efficiently and improve morbidity reduction by appropriately monitoring patients at risk.

With the rapid development of software, there is increasing use of machine learning algorithms. Especially, machine learning methods have been applied in medicine with excellent results, deriving predictive algorithms for multiple conditions (8–15). While traditional predictive models employ selected parameters, machine learning methods easily include multiple clinical parameters (16).

Although some predictive score systems or risk calculators have been developed by previous studies for the prediction of pulmonary complications (3, 5, 9, 13, 17), to date, studies assessing RF feature selection and machine learning algorithms are rare in the M-STBI population.

We hypothesized that supervised machine learning could help to develop models for better predicting single ARF occurrence upon M-STBI compared with routine statistical models. Therefore, this study aimed to utilize a machine learning model for developing and validating an ARF predictive model, termed extreme gradient boosting (XGBoost), which was compared to a conventional logistic regression model for effectiveness.

## Materials and Methods

### Data Source

Model development and internal validation were based on a large TBI database, which consists of data of patients admitted to the department of neurosurgery in the Second Affiliated Hospital of Fourth Military Medical University, China, from September 2015 to May 2017. This trial had approval from the Institutional Ethics Board of the Second Affiliated Hospital of Fourth Military Medical University (TDLL-KY-202110-09) and data reporting followed the guidelines included in the Transparent Reporting of a multivariable prediction model for Individual Prognosis Or Diagnosis (TRIPOD) statement (18).

### Selection of Patient

Adult patients with M-STBI [3 ≤ Glasgow Coma Scale (GCS) ≤ 12] with a definite history of brain trauma and abnormal head on CT images, acquired from September 2015 to May 2017, were included in this study. Patients with age >80 years or <18 years, multiple injuries with TBI upon admission, or pregnancy (in women) were excluded from this study.

### Data Collection and Outcome Definition

The medical records of the patients were carefully collected by three authors on separate occasions. Demographic parameters, clinical and laboratory variables, comorbidities, imaging features, and outcome variables were recorded. All the patients with M-STBI underwent the procedure of arterial blood gas (ABG) analysis within the day of admission; ABG was repeated, if oxygen saturation (SpO_2_) < 93% using a nasal catheter or mask oxygen inhalation for at least 5 min after suctioning oropharyngeal secretions. The primary endpoint of this study was ARF within 72 h of admission, which was defined as respiratory failure with partial pressure of oxygen (pO_2_) <60 mm Hg and respiratory rate >30 breaths/min or respiratory distress for at least 5 min (19).

### Predictors of ARF

Clinical and laboratory parameters recorded in the initial 48 h after ICU admission were examined for their capabilities of predicting ARF. For parameters measured many times, both the maxima and minima were examined. Age, gender, GCS, comorbidity, and imaging features including the Marshall CT score and severity scores of lung exudations (see Tables S1, S2 for the details of the scores) were analyzed. In addition, laboratory data such as white blood cell (WBC) and neutrophil counts, neutrophil–lymphocyte ratio, C reactive protein (CRP), and procalcitonin (PCT) were included. In term of therapy, long-term sedation (sedation duration > 48 h) was examined. For predictor selection, the Akaike Information Criterion (AIC) was used for minimizing the possible collinearity of parameters from a given patient as well as overfitting (20).

This was a hypothesis-generating retrospective trial, with no sample size estimation, but including the totality of eligible patients in the database for statistical power maximization.

### Missing Data

We aimed to reflect daily clinical routine where often not all the data are obtainable. To make our algorithms and study realistic, we decided not to correct for missing data, e.g., by imputation techniques and to perform the analysis using the available data only. While using imputation techniques to estimate missing variables have many merits in conventional statistics, it is less preferred in machine learning because it does not reflect the observed reality—at best a close approximation—and adds artificially introduced noise to the data. Moreover, there could be significant reasons why some data are missing, which could be linked to the outcome variable of interest. In such cases (and in a number of other scenarios), imputation obscures important relationships in the observed data or introduces artificial relationships altogether, which decreases the value of complex pattern recognition used in machine learning. For variables with missing values, we coded the missing value as zero and created the corresponding missing dummy (12).

### Statistical Analysis

Continuous and categorical data were presented as median [interquartile range (IQR)] and number (percentage). Demographic characteristics between participants with and without ARF were compared by the Mann–Whitney *U* test or the chi-squared test.

The primary model of this study was the XGBoost gradient boosted tree model. XGBoost represents a tree ensemble technique building in a progressive fashion on the loss from weak decision tree base learners. It can learn rapidly and effectively from substantial data amounts, with a flexibility allowing learning even from missing data (21). After tuning the XGBoost model, parameters of the XGBoost model were finally max_depth = 7, subsample = 0.94, colsample_bytree = 0.83, nrounds = 100, learning rate (eta value) = 0.3, and gamma = 5.

For comparison, another model for predicting ARF occurrence was developed based on the multivariate logistic regression analysis.

As a comparison, a second model to predict the occurrence of ARF was created using the multivariate logistic regression model.

For comparison, model discrimination was assessed using the area under the receiver operating characteristic (AUROC) curve and the optimal cutoff value was calculated by Youden index. The confusion matrixes of the two models were created based on the optimal cutoff values to evaluates the accuracy, sensitivity, and specificity.

EmpowerStats (X&Y Solutions, Inc., Boston, MA, USA) and R version 3.4.2 (http://www.R-project.org) were utilized for data analysis. *p* < 0.05 was considered as statistically significant.

## Results

### Patients

**Figure 1:**
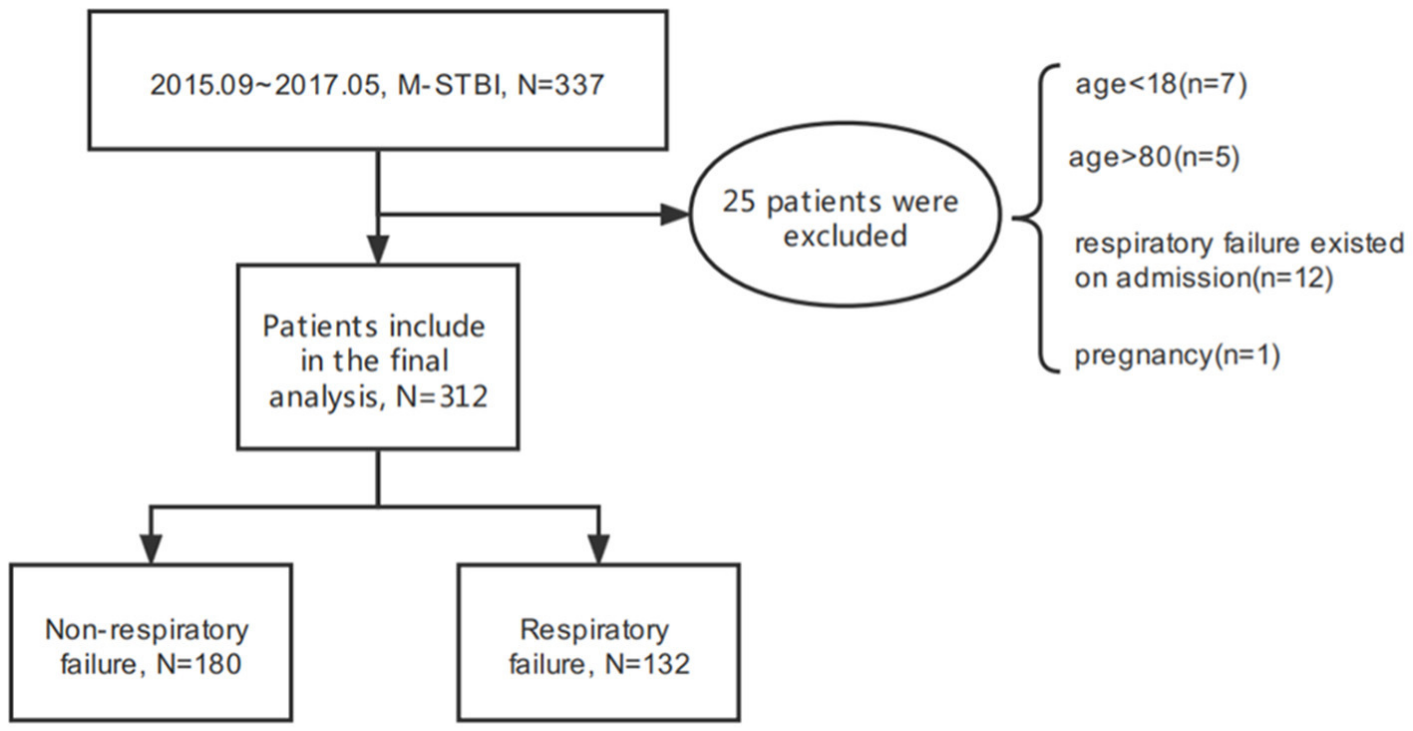
Study flowchart.

Between September 2015 and May 2017, 312 M-STBI cases hospitalized in the non-ICU (NICU) of the Second Affiliated Hospital of Fourth Military Medical University were examined. There were 232 males and 80 females. Of all patients, 132 (42.3%) patients had ARF (Figure 1). Characteristics of patients are given in detail in Table 1.

**Table 1:** Baseline characteristics of patients with moderate-to-severe traumatic brain injury with or without acute respiratory failure (ARF).

**Exposure**	**Non-ARF (*N* = 180)**	**ARF (*N* = 132)**	***P*-value**
AGE	56.0 (45.8–66.0)	57.0 (49.0–66.0)	0.448
Sex (male/%)	129 (71.67%)	103 (78.03%)	0.203
Smoking	48 (26.67%)	49 (37.12%)	0.049
GCS	8.0 (6.0–11.0)	6.0 (5.0–8.0)	<0.001
Marshall score	5.0 (4.0–6.0)	6.0 (5.0–6.0)	<0.001
Scores of lung exudations			0.004
0	46 (25.56%)	16 (12.12%)	
1	14 (7.78%)	6 (4.55%)	
2	120 (66.67%)	110 (83.33%)	
Comorbidity			
Hypertension (*n*, %)	92 (51.11%)	69 (52.27%)	0.839
Diabetes	12 (6.67%)	7 (5.30%)	0.619
COPD	2 (1.11%)	7 (5.30%)	0.029
Cardiovascular disease	3 (1.67%)	7 (5.30%)	0.072
Long-term sedation	75 (41.67%)	102 (77.27%)	<0.001
White cell count, × 10^9^/L	10.3 (7.4–13.7)	13.6 (10.3–18.4)	<0.001
Neutrophil cell count, %	81.3 (73.2–87.0)	87.5 (82.5–90.7)	<0.001
Neutrophil-lymphocyte ratio	7.3 (3.0–11.6)	4.4 (1.4–8.0)	<0.001
CRP, mg/L	6.2 (5.0–28.0)	26.9 (5.0–97.7)	<0.001
Not recorded, n	104 (57.7%)	69 (52.3%)	
PCT, ng/mL	0.2 (0.2–0.3)	0.3 (0.2–1.7)	<0.001
Not recorded, n	96 (53.3%)	54 (40.9%)	
LOH	10.0 (7.0–16.0)	17.5 (11.0–28.0)	<0.001

*Data are expressed as medians ± interquartile ranges and n (percentage), as appropriate*.

*GCS, Glasgow Coma Scale; COPD, chronic obstructive pulmonary disease; CRP, C-reactive protein; PCT, procalcitonin*.

The ARF group included more individuals with smoking history (37.12 vs. 26.67%; *p* = 0.049) and chronic obstructive pulmonary disease (COPD) history (5.30 vs. 1.11%; *p* = 0.029) prior to ICU admission than the non-ARF group. Upon admission, the minimum GCS values (6.57 ± 2.68 vs. 8.63 ± 3.27 mmol/l; *p* < 0.001) were lower, while the Marshall CT scores (5.50 ± 0.95 vs. 4.70 ± 1.39; *p* < 0.001) and severity scores of bilateral lung exudations (83.33 vs. 66.67%; *p* = 0.004) were higher in ARF cases. ARF cases also showed elevated white blood cell count (14.87 ± 7.14 vs. 10.96 ± 5.16; *p* < 0.001), elevated neutrophil cell count (85.06 ± 9.47 vs. 78.27 ± 12.37; *p* < 0.001), lower neutrophil–lymphocyte ratio (5.66 ± 5.83 vs. 9.78 ± 10.11; *p* < 0.001), and higher CRP (57.10 ± 59.85 vs. 23.51 ± 31.19 mmol/l; *p* < 0.001) and PCT (2.54 ± 6.09 vs. 0.42 ± 1.13; *p* = 0.002) compared with the non-ARF group (Table 1).

### The XGBoost Model

Extreme gradient boosting had an AUROC of 0.84 in the training set, with sensitivity and specificity of 0.71 and 0.84, respectively. Its precision was 0.78 (95% CI: 0.72–0.83). An error rate of 0.12 was obtained, indicating a correct prediction in roughly 78% of patients (Table 2).

**Table 2:** The multivariate logistic regression model with stepwise variable selection.

**Variables**	**OR (95% CL)**	***P-*value**
Smoking	2.092 (0.989–4.429)	0.054
Scores of lung exudations 1	0.988 (0.158–6.172)	0.990
Scores of lung exudations 2	3.435 (1.248–9.456)	0.017
WBC	1.078 (1.012–1.148)	0.020
GCS	0.788 (0.681–0.913)	0.002
Marshall score	1.706 (1.181–2.463)	0.004
Long-term sedation	6.293 (2.908–13.621)	0.001
PCT	1.121 (0.924–1.360)	0.249
CRP	1.014 (1.004–1.025)	0.007
^a^PCT (not recorded)	0.540 (0.245–1.190)	0.126

*WBC, white blood cell; GCS, Glasgow Coma Scale; COPD, chronic obstructive pulmonary disease; CRP, C-reactive protein; PCT, procalcitonin*.

a *PCT dummy variable for missing values*.

In the test population, an AUROC of 0.90 was obtained for XGBoost, which had specificity and sensitivity of 0.85 and 0.78, respectively, indicating correct prediction of 29 of the 37 ARF cases in the test set. Meanwhile, 8 cases were incorrectly predicted [reflecting a precision rate of 0.82 (0.72, 0.90)]. The model had an error rate of 0.18, indicating correct outcome prediction in >81% of cases (Table 2).

**Figure 2:**
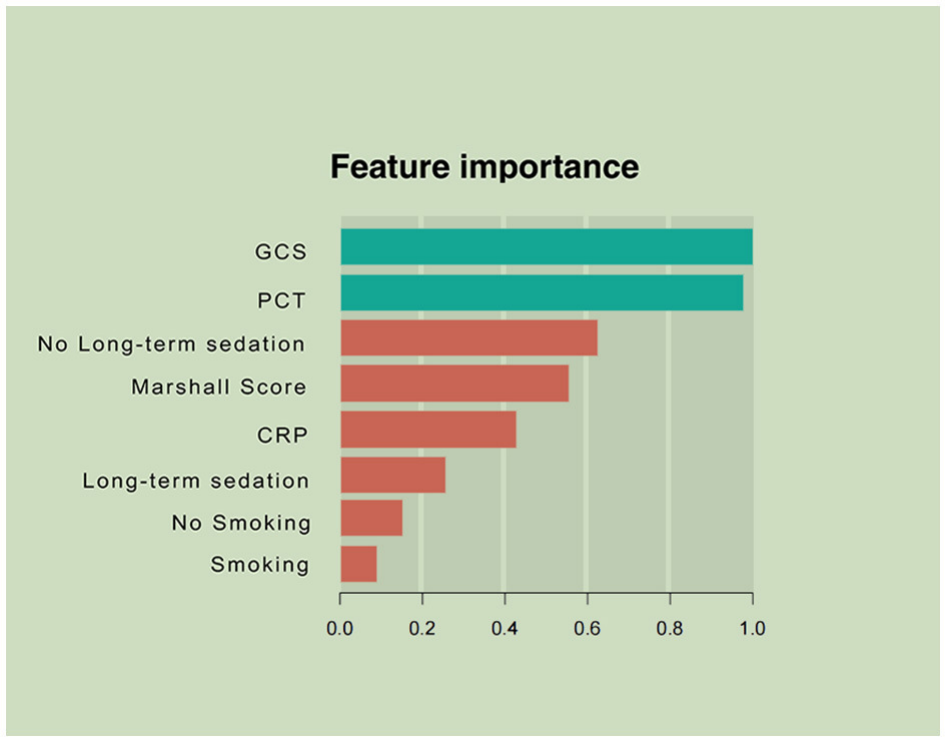
Parameters by predictive value in the extreme gradient boosting (XGBoost) model. To predict acute respiratory failure (ARF) following moderate or severe traumatic brain injury, gradient boosting used various variables based on their importance in prediction modeling. In this analysis, the Glasgow Coma Scale (GCS) and inflammation-associated laboratory parameters upon admission had higher values in ARF prediction than other features of patient.

Variables showing high predictive values were the GCS and the Marshall CT score, PCT, and CRP on admission. The GCS was the center factor of the XGBoost model because the gain of the GCS was the highest among all the variables (Figure 2). Other variables, e.g., long-term sedation and smoking history had low prediction power (Figure 2).

### Logistic Regression Model

Baseline parameters for the ARF and non-ARF groups are shown in Table 1. Smoking and COPD history, the GCS and the Marshall CT score on admission, severity scores of lung exudations, long-term sedation, neutrophil cell count, WBC, neutrophil–lymphocyte ratio, PCT, and CRP showed associations with ARF occurrence in the univariate analysis (*p* < 0.05, Table 1). In the stepwise multivariate logistic regression analysis, bilateral lung exudations [odds ratio (OR), 3.435; 95% CI, 1.248–9.456], the Marshall CT score (OR for each 1 score increase, 1.078; 95% CI, 1.012–1.148), long-term sedation, increased WBC (OR for each 1 × 10^9^/L increase, 1.076; 95% CI, 1.181–2.463), and CRP (OR for each 1 mg/l increase, 1.014; 95% CI, 1.004–1.025) were associated with increased probability of ARF. On the contrary, the GCS (OR for each 1 score increase, 0.788; 95% CI, 0.681–0.913) was associated with decreased probability of ARF (Table 2).

The multivariate regression model was created based on the AIC-selected variables. It showed an AUROC of 0.943 in the training cohort, with a specificity of 0.946 and a sensitivity of 0.837 (Table 3). Its error rate was 11.6%. In the test population, AUROC was 0.792 and specificity and sensitivity were 0.913 and 0.667, respectively; its error rate approximated 15.6% (Table 3).

**Table 3:** Confusion matrix for machine learning.

**Training data**				**Statistical analysis**	
Predicted true	0	1	Total	AU-ROC	0.840
0	112	28	140	Accuracy	0.782
1	22	67	89	Sensitivity	0.705
Total	134	95	229	Specificity	0.836
Test data				Statistical analysis	
Predicted true	0	1	Total	AU-ROC	0.902
0	39	8	47	Accuracy	0.820
1	7	29	36	Sensitivity	0.784
Total	46	37	83	Specificity	0.848

*AU-ROC, area under the receiver operating characteristic*.

### Model Performances

**Figure 3:**
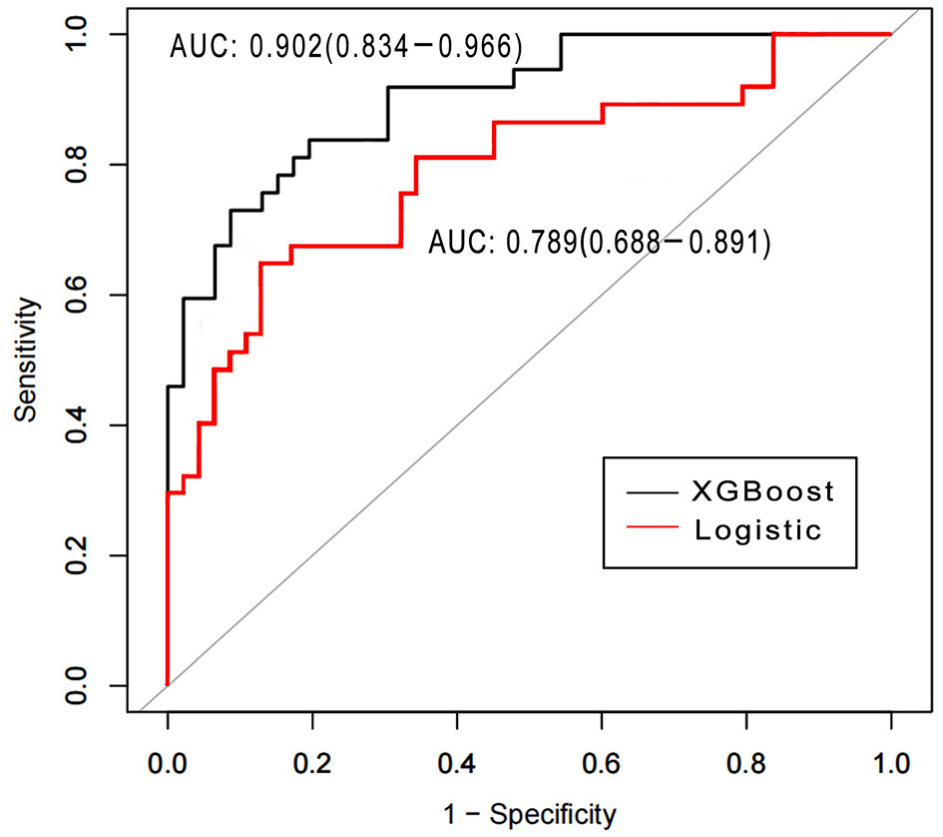
Receiver operating characteristic curves for examining the discriminative powers of the XGBoost and the logistic regression models.

Area under the receiver operating characteristics were determined for assessing the discriminative abilities of both the models. XGBoost showed an elevated AUROC in comparison with the logistic regression model (AUROC, 0.902; 95% CI, 0.834–0.966 vs. 0.789; 95% CI, 0.688–0.891, *p* < 0.05; Figure 3). Tables 3, 4 describe the classification and confusion matrixes for both the models in predicting ARF.

**Table 4:** Confusion matrix for conventional statistics.

**Training data**				**Statistical analysis**	
Predicted true	0	1	Total	AU-ROC	0.879
0	99	12	111	Accuracy	0.795
1	35	83	118	Sensitivity	0.874
Total	134	95	229	Specificity	0.739
Test data				Statistical analysis	
Predicted true	0	1	Total	AU-ROC	0.789
0	40	13	53	Accuracy	0.771
1	6	24	30	Sensitivity	0.649
Total	46	37	83	Specificity	0.870

*AUROC, area under the receiver operating characteristic*.

## Discussion

Prediction and timely detection of ARF in patients with M-STBI are critical, crucially impacting M-STBI outcome (22, 23). This study developed a machine learning-based model to predict ARF occurrence in M-STBI, with multiple remarkable features. First, the model included readily available and reproducible parameters in the initial 48 h after admission. Second, after analyzing multiple interaction patterns among variables, the predominance of admission-related parameters (the GCS and the Marshall CT score, CRP, PCT, and long-term sedation; Figure 2) was most significant in determining the occurrence of ARF. Third, the novel model enhanced performance compared with the conventional logistic regression model.

This study first investigated ARF prediction in patients with M-STBI using machine learning methods. This new model had accuracy and AUROC of 0.83 and 0.90, respectively. Of greatest importance, sensitivity and specificity of 0.73 and 0.91, respectively, were obtained in the test cohort.

First, accurate detection of ARF in critically ill individuals with M-STBI is essential in performing intensive airway management and making decision with respect to invasive treatments such as tracheal intubation, invasive mechanical ventilation, and even tracheostomy. To date, reliable tools for timely predicting ARF in M-STBI are lacking. In this study, we demonstrated enlightened machine learning methods, including XGBoost, could provide a great deal of information obtainable from databases and promote the development and validation of better predictive models in comparison with conventional logistic regression techniques. The new model could help to stratify M-STBI cases right after ICU admission. Therefore, intensive airway management or invasive treatment could be more accurately provided to individuals with high odds of developing ARF to avoid long-term hypoxia, which is associated with increased morbidity and mortality in patients with M-STBI (24, 25). On the other hand, intensive airway management needs important human and material resources, while invasive treatment is related to complications and high medical costs. Thus, identifying individuals who could benefit from intensive airway management or invasive treatment are critical. However, this analysis provided no high level of evidence with respect to the effectiveness of XGBoost. Further randomized controlled trials that compare therapies dependent on and independent of the predictive model should comprehensively examine its effectiveness.

Second, we aimed to design a model with easy implementation by neurosurgery residents and staff alike. Therefore, parameters easily available and reproducible upon admission were required and quantitative (blood test results, the GCS score, the Marshall CT score, etc.) and dichotomous (long-term sedation or not, smoking status, etc.) variables were selected.

Third, the XGBoost model showed that the GCS score, PCT, the Marshall CT score, CRP, and long-term sedation potentially predicted ARF in patients with M-STBI. Consistent with previous reports, the GCS score, PCT, and CRP were related to ARF in patients with M-STBI, suggesting the extent of TBI and severity of systematic inflammation (26–29). The GCS was center factor in the XGBoosting model shown in Figure 2, suggesting that the severity of brain injury was associated with ARF in patients with M-STBI significantly. The results agreed with clinical experience very well. However, to the best of our knowledge, the association between the Marshall CT score and ARF has not been confirmed. This study suggested that the Marshall CT score potentially predicted ARF. The explanation could be that the Marshall CT score can reflect the extent of brain injury based on neuroimaging, so the high Marshall CT score is associated with injury of brainstem centers of respiration or intracranial hypertension, which causes ARF easily. Moreover, both the logistic and XGBoost models showed that sedation (more than 48 h) was related to ARF. The results could be explained by the fact that sedation is an important tool for reducing intracranial pressure, which cannot be stopped until intracranial pressure returns to normal. Intracranial hypertension and respiratory depression caused by sedative drugs contribute to ARF (30, 31).

This study had many strengths. XGBoost modeling represents a new method not yet applied in respiratory failure studies of neurological critical patients. XGBoost modeling can learn swiftly with high efficiency from important data amounts and its high flexibility enables learning even from missing data (21). The XGBoost model had starkly higher predictive accuracy compared with the generalized linear model, being capable of capturing complex associations in data without requiring explicit high-order interactions and non-linear functions (12). Using such features, predictive models based on clinical and laboratory variables, which are easily available and reproducible upon admission, could be built. However, there were also limitations. First, as a hypothesis-generating study, external validation of the XGBoost model is important for confirming its usefulness. The XGBoost model developed in this study will be applied to the Medical Information Mart for Intensive Care (MIMIC)-IV for external validation in the next study. Second, because this was a retrospective study, missing data are inevitable in practice. For missing data, variables with >70% missing values were excluded from model construction. Thus, the sample sizes of the training (*n* = 86) and test (*n* = 32) sets were low especially in the logistic regression model. To some extent, missing data decreased the performance of the model. Third, this study only explored ARF within 48 h upon admission and a different time interval (e.g., >48 h following admission) was not studied.

## Conclusion

In total, six major parameters related to ARF were screened to develop the XGBoost model with enhanced predictive value for ARF compared with the logistic regression model in patients with M-STBI.

## Data Availability Statement

The raw data supporting the conclusions of this article will be made available by the authors, without undue reservation.

## Ethics Statement

The studies involving human participants were reviewed and approved by Institutional Review Board of the Second Affiliated Hospital, Fourth Military Medical University. Written informed consent for participation was not required for this study in accordance with the national legislation and the institutional requirements.

## Author Contributions

ML and RM contributed to the study conception, design, and manuscript drafting. YH, MC, FB, and ZS contributed to the acquisition of data and analysis and interpretation of data. All authors approved the final version of the manuscript.

## Funding

This study was funded by the Youth Innovation Fund of the Second Affiliated Hospital of Fourth Military Medical University (Grant No. 2021LCYJ026).

## Conflict of Interest

The authors declare that the research was conducted in the absence of any commercial or financial relationships that could be construed as a potential conflict of interest.

## Publisher's Note

All claims expressed in this article are solely those of the authors and do not necessarily represent those of their affiliated organizations, or those of the publisher, the editors and the reviewers. Any product that may be evaluated in this article, or claim that may be made by its manufacturer, is not guaranteed or endorsed by the publisher.
