# Supplementary material for: Machine Learning Model for Predicting Acute Respiratory Failure in Individuals With Moderate-to-Severe Traumatic Brain Injury
Source: Front Med (Lausanne). 2021 Dec 24;8:793230. doi: 10.3389/fmed.2021.793230 (PMC8739486; doi:10.3389/fmed.2021.793230)
Supplement: Supplementary file 1 [file Table_1.DOCX]

Supplementary Material

# Supplementary Tables

Table S1. Marshall CT scan score

| **Score** | | **Definition** |
| --- | --- | --- |
| 1 | No visible intracranial pathology seen on CT scan | |
| 2 | Cisterns are present with midline shift of 0–5 mm and/or lesions densities present;  no high- or mixed-density lesions >25 cm^3^ may include bone fragments and foreign bodies | |
| 3 | Cisterns compressed or absent with midline shift of 0–5 mm;  no high- or mixed-density lesions >25 mm | |
| 4 | Midline shift >5 mm; no high- or mixed-density lesions >25 cm^3^ | |
| 5 | Any lesion surgically evacuated | |
| 6 | High- or mixed-density lesions >25 cm^3^; not surgically evacuated | |

Table S2. Severity scores of exudations in lung

| Classification | Description |
| --- | --- |
| 0 | no exudations in lung |
| 1 | unilateral exudative in lung |
| 2 | bilateral exudative in lung |
